# P-1196. Geographic distribution, MIC testing, and susceptibility rates of Candida auris isolates collected in the United States since 2014

**DOI:** 10.1093/ofid/ofaf695.1389

**Published:** 2026-01-11

**Authors:** Marisa Winkler, John Kimbrough, Kelley A Fedler, Samuel Edeker, Abby Klauer, Paul Rhomberg, Mariana Castanheira

**Affiliations:** Element Materials Technology/Jones Microbiology Institute, North Liberty, Iowa; Element Iowa City (JMI Laboratories), North Liberty, Iowa; Element, North Liberty, Iowa; Element Materials Technology/Jones Microbiology Institute, North Liberty, Iowa; JMI Laboratories, North Liberty, Iowa; Element Materials Technology/Jones Microbiology Institute, North Liberty, Iowa; Element, North Liberty, Iowa

## Abstract

**Background:**

*Candida auris* (CARS) is an emerging infectious threat due to rising infection rates, eradication difficulty, and multidrug resistance. The US Centers for Disease Control (CDC) has tentative resistant (R)-only breakpoints (BP) against CARS for fluconazole (FLC), amphotericin B (AmB), anidulafungin (AND), caspofungin (CAS), and micafungin (MCF) with the caveat that the correlation between the values and clinical outcomes is unknown. Susceptible (S)-only BP for CARS from the Clinical and Laboratory Standards Institute (CLSI) exist for rezafungin (RZF). The CLSI S-only BP for RZF is several dilutions lower than the R CDC BPs for other echinocandins (ECH). It is imperative to understand the geographic distribution of CARS and MIC patterns to RZF and other agents.

**Figure:**
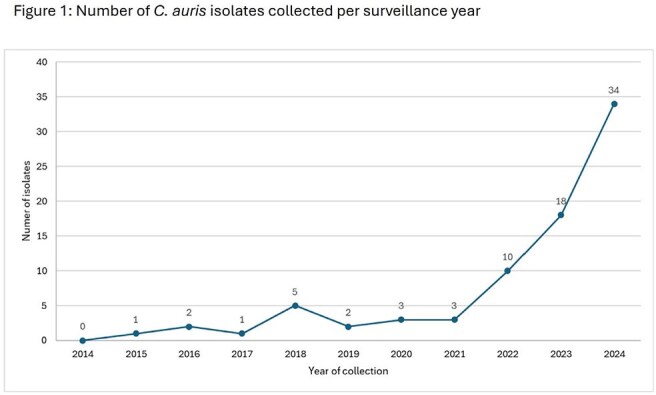

**Figure:**
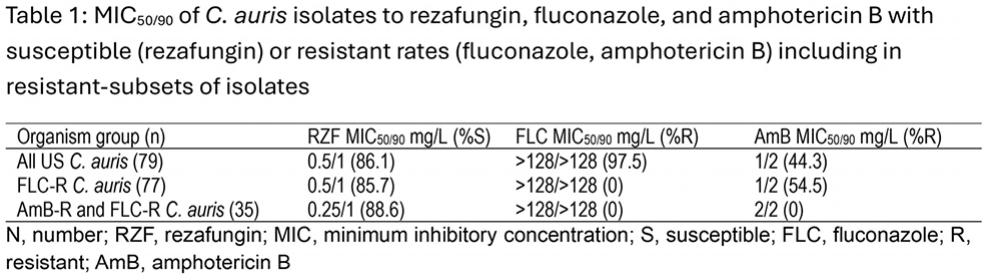

**Methods:**

CARS clinical isolates were collected from invasive candidal infections between 2014 – 2024 in 12 hospitals in 5 US Census divisions. Antifungal susceptibility testing was performed by reference broth microdilution with interpretation for AND, CAS, MCF, FLC, and AmB by CDC criteria (R ≥4, ≥2, ≥4, ≥32, and ≥2 mg/L) and RZF by CLSI criteria (NS≥1 mg/L). Isolates R/NS to any ECH were submitted to whole genome sequencing and analyzed for alterations in FKS1.

**Figure:**
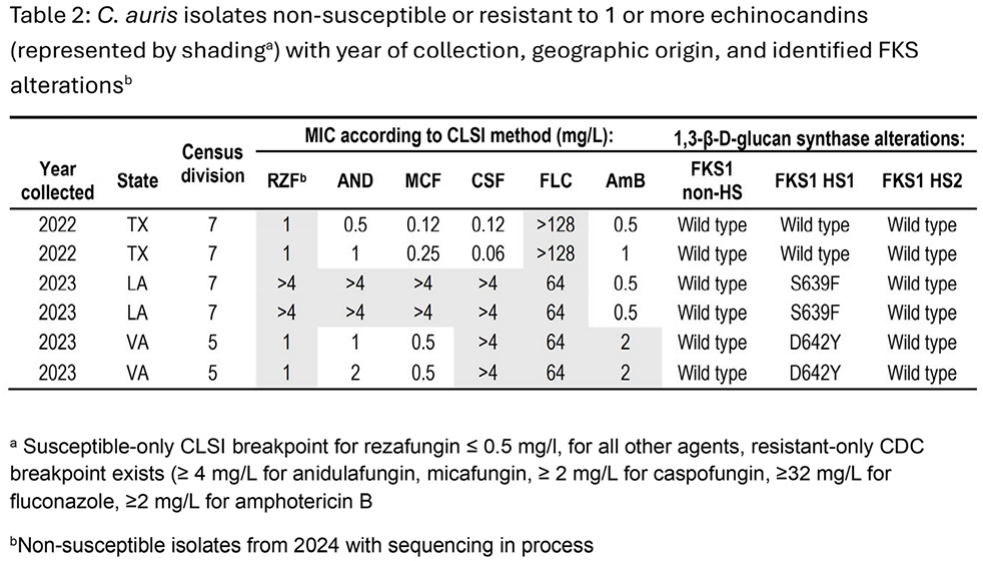

**Results:**

79 isolates were tested. Exponential increase in numbers is seen since 2021 (Figure 1). 86.1% were S to RZF; 97.5% and 44.3% were R to FLC and AmB, respectively. 77 isolates were R to FLC, within this group, 85.7% were S to RZF (Table 1). 35 isolates were R to AmB, these were all R to FLC and 88.6% were S to RZF. No RZF NS isolates were collected prior to 2022, there were 2 isolates in 2022, 4 in 2023, and 5 in 2024 (11 total). Isolates that were only NS to RZF were wildtype (WT) for FKS1 whereas those R/NS to ≥ 1 ECH had FKS1 alterations (Table 1).

**Conclusion:**

CARS isolates have been rapidly increasing in prevalence-based surveillance. For ECH, molecular analysis indicates that isolates with an *in vitro* susceptibility profile showing NS to RZF but not-R to other ECH are WT for FKS1. RZF is an empiric treatment of choice for CARS given high R rates to FLC and AmB, minimal cross-resistance, and lack of S breakpoints for other ECH.

**Disclosures:**

Marisa Winkler, MD, PhD, Basilea: Advisor/Consultant|Basilea: Grant/Research Support|GSK: Advisor/Consultant|GSK: Grant/Research Support|Melinta Therapeutics: Advisor/Consultant|Melinta Therapeutics: Grant/Research Support|Mundipharma: Advisor/Consultant|Mundipharma: Grant/Research Support|Pfizer: Advisor/Consultant|Pfizer: Grant/Research Support|Pulmocide: Advisor/Consultant|Pulmocide: Grant/Research Support Kelley A. Fedler, BS, Melinta Therapeutics: Grant/Research Support Mariana Castanheira, PhD, Melinta Therapeutics: Advisor/Consultant|Melinta Therapeutics: Grant/Research Support

